# Automatic recognition of anatomical structures and surgical phases in robot-assisted minimally invasive esophagectomy (RAMIE) using deep learning: a retrospective cohort study

**DOI:** 10.1007/s00464-026-12985-1

**Published:** 2026-07-09

**Authors:** Romy C. van Jaarsveld, Yiping Li, Ronald L. P. D. de Jong, Franco Badaloni, Gino M. Kuiper, Tim J. M. Jaspers, Marcel Breeuwer, Fons van der Sommen, Richard van Hillegersberg, Yasmina Al Khalil, Jelle P. Ruurda

**Affiliations:** 1 Department of Surgery, University Medical Center Utrecht, Heidelberglaan 100, 3584 CX Utrecht, The Netherlands; 2 Department of Biomedical Engineering, Eindhoven University of Technology, Eindhoven, The Netherlands; 3 Department of Electrical Engineering, Eindhoven University of Technology, Eindhoven, The Netherlands

**Keywords:** Robotic esophagectomy, Artificial intelligence, Deep learning, Surgical computer vision, Anatomy segmentation, Surgical phase recognition

## Abstract

**Background:**

Curative treatment of resectable esophageal cancer comprises neoadjuvant chemoradiotherapy and esophagectomy. Robot-assisted minimally invasive esophagectomy (RAMIE) is the preferred technique; however, learning RAMIE is challenging due to the complex chest anatomy, patient positioning and zoomed-in camera view. Computer-aided anatomy recognition holds promise for improving surgical navigation. This study aims to develop real-time anatomy recognition and surgical phase recognition algorithms for the thoracic part of RAMIE using deep learning and to understand the challenges of current state-of-the-art algorithms.

**Methods:**

A retrospective single-center cohort study was conducted on prospectively collected RAMIE videos at University Medical Center Utrecht, The Netherlands. Two datasets were created: an anatomy segmentation dataset with 1504 frames from 53 videos, annotated for eight anatomical classes and four surgical instruments; and a surgical phase dataset, with 38 videos labeled with thirteen distinct phases. Several deep learning models were trained and tested using both datasets.

**Results:**

The SegNeXt model achieved the most accurate segmentations with an overall overlap score of 0.72 for all classes, of which the surgical instruments were best detectable. The best surgical phase recognition model achieved an overall accuracy of 82.8% and revealed loss of accuracy during phase transitions. Class imbalance affected both datasets which led to less frequent appearing classes performing lowest.

**Conclusions:**

This study identified suitable models for computer-aided, real-time anatomy and surgical tool segmentation and surgical phase recognition for the thoracic part of RAMIE. Acceptable performance was achieved while both datasets are highly complex. Performance is expected to improve by further expanding and diversifying the datasets.

**Graphical abstract:**

**Figure:**
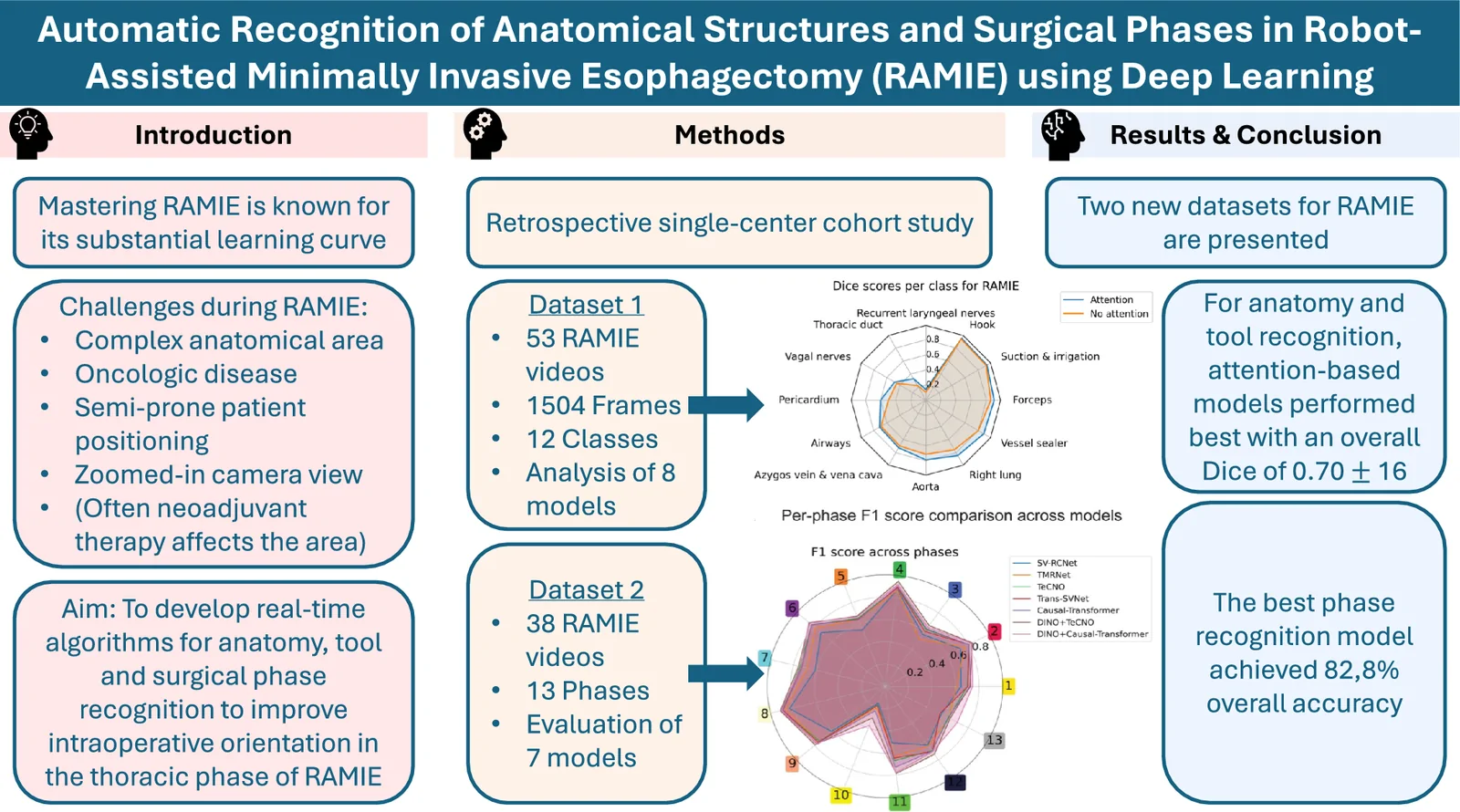

**Supplementary Information:**

The online version contains supplementary material available at https://doi.org/10.1007/s00464-026-12985-1.

Esophageal cancer is the 11^th^ most common cancer worldwide and ranks 7^th^ in oncologic mortality [1]. Curative treatment comprises neoadjuvant chemoradiotherapy and esophagectomy with two-field lymphadenectomy [2]. Robot-Assisted Minimally Invasive Esophagectomy (RAMIE) is increasingly utilized as it offers advantages such as an enlarged three-dimensional view, articulated instruments, and improved ergonomics [3, 4]. RAMIE is particularly beneficial during the thoracic phase, where enhanced visualization and instrument precision allow for more accurate dissection in the narrow mediastinal working space, leading to improved thoracic lymphadenectomy outcomes [5]. The unique peri-esophageal anatomy is delicate, specifically the surrounding airways, nerves, lymphatics and blood vessels. In these patients, the complex area is additionally affected by oncologic disease and neoadjuvant radiotherapy. Combining this with the semi-prone patient positioning required in RAMIE and the zoomed-in camera view leads to RAMIE being a challenging surgery to perform. To master RAMIE, the learning curve ranges from 20 to 80 cases, in which recognition of anatomy plays a major role [6, 7]. Assisting surgeons with intraoperative guidance could improve vital structure detection, surgical orientation and surgeons’ confidence [8, 9]. For surgeons in training, it could accelerate their learning curve [6]. Improved orientation could prevent iatrogenic complications, thus improving perioperative surgical outcomes.

Information on surgical phases and the respective anatomy that is expected to be encountered could help the surgeon’s intraoperative orientation and could improve their progress during the procedure. This study aims to develop real-time deep learning algorithms for anatomy and surgical phase recognition in the thoracic part of RAMIE, focusing on multiple key anatomical structures, as well as to understand challenges and limitations of current state-of-the-art algorithms.

## Materials and methods

A retrospective single-center cohort study was conducted at University Medical Center (UMC) Utrecht, The Netherlands. Ethical approval was waived. Operation recordings were prospectively collected of all patients who underwent transthoracic RAMIE for esophageal cancer, from January 2018 to July 2021. The only exclusion criterion was no thoracic recording available. A preliminary study demonstrated the potential for anatomy recognition of the azygos vein and vena cava, aorta and right lung in manually selected frames [10]. To better fit the aim of developing real-time recognition algorithms that could function during live surgery, for this study, videos were selected from the database at chance, avoiding selection bias. The patient group was treated with or without neoadjuvant chemoradiotherapy as decided by a multidisciplinary team and operated on by expert robotic upper-gastrointestinal surgeons using da Vinci surgical systems (Intuitive Surgical Inc., Sunnyvale, CA, USA). The focus of this study was on the thoracic phase, until the division of the esophagus. Additional information on model training and evaluation can be found in the appendix.

Two datasets were created: an anatomy segmentation dataset [11] for anatomy recognition, and a surgical phase recognition dataset [12], which can provide additional information by recognizing which anatomical structures appear in the different phases. Both datasets were labeled by two research fellows (PhD candidates) trained for RAMIE anatomy and surgical phase recognition, supervised by an expert surgeon. Subsequently, the expert surgeon reviewed all test set annotations and corrected any remaining errors made by the research fellows. To evaluate annotation quality, a subset was compared with annotations made independently by an expert surgeon, which served as the reference standard. All annotations were performed manually; no machine learning pipeline was used in the creation of the datasets. While annotations by multiple experts could strengthen the dataset, the expert’s experience and the fellows’ training ensure high-quality annotations. Future work may include annotations by additional experts.

### Dataset 1—anatomy and surgical tool recognition

For dataset 1, 1504 frames were selected from 53 edited RAMIE videos. The editing process consisted of removing non-informative visual elements, such as black borders and the user interface of the robot. In total, 75% of the data was obtained through random sampling, which led to underrepresentation of certain anatomy classes. To address this, the remaining 25% was manually selected to ensure adequate representation of minority classes. The selection process was blinded to model outputs. Twelve classes were annotated, including four surgical instruments: cadiere forceps, cautery hook, vessel sealer, and suction/irrigation system. Segmenting the surgical tools may improve anatomical recognition by accounting for tool-tissue occlusions. Furthermore, it lays the groundwork for future tool-to-tissue distance tracking. Eight anatomical structures were annotated: airways (trachea and main bronchi), aorta, azygos vein, superior vena cava, vagal and recurrent laryngeal nerves, thoracic duct, pericardium and right lung, serving as landmarks for lymphadenectomy and esophagectomy. The azygos vein and superior vena cava were combined into a single class due to their similar appearance and indistinct boundary.

An annotation protocol was established by an experienced surgeon, postdoc, and research fellows. The aim of the protocol was to create a standardized workflow to create a dataset that is both clinically relevant and technically usable. As no consensus for anatomy segmentations in RAMIE exists, the protocol was refined according to our own experience, interdisciplinary meetings with study groups working on similar surgical datasets and literature on anatomy segmentation in other surgical fields. During the annotation process, quantitative and qualitative feedback was regularly collected through intermittent survey studies among upper-gastrointestinal surgeons of varying experience, leading to changes in the protocol and adjustments in the dataset accordingly. Minor changes included grouping the trachea and bronchi into one class, grouping the pulmonary veins and pericardium into one class and including cauterized tissue within the boundaries of segmentations. One major change in our protocol was the decision to exclude the esophagus from the dataset. The appearance of the esophagus during dissection and variability of its location in the surgical field due to the manipulation of the surgeon led to low performance scores and surgeons did not see the added value of a segmented esophagus during RAMIE. More recent survey studies confirmed the value of intraoperative anatomy recognition in RAMIE [8, 13] and no refinements to the dataset were needed. The basis of the protocol was to annotate what is visible. Anatomical structures or tools that are behind something else are not annotated. Our reasoning behind this rule is, if we cannot see it, a model will not be able to properly predict it as well. For anatomy segmentations, we first defined the boundaries of all structures based on what is anatomically known and translated that to the RAMIE frames based on their location, color and shape. Included in the boundaries were cauterized tissue, connective and fatty tissue, blood and smoke that did not obscure the boundary. Excluded were clips, as used on the azygos vein and thoracic duct, and occlusions that obscured the boundary of the structure. For example, a blood clot in the middle of the structure was included in a segmentation, but a blood clot obscuring a boundary which would have led to guessing where the boundary would be was excluded from the segmentation. The thoracic duct was the only exception to these rules. During RAMIE, the wall of the duct is very rarely visible. The duct is dissected together with the surrounding fatty tissue to prevent damage to the wall. For our segmentations, this led to annotating the fatty tissue around the thoracic duct, which was recognizable because of its consistent location. Consensus on tool segmentation was more easily reached as they have clear boundaries and were delineated as such. Openings were not included in the segmentation, as present in forceps and graspers. Regarding occlusions, the same rules as for the anatomy segmentations were applicable.

For each frame, all visible anatomical structures and instruments were manually annotated using click-based segmentation in Label Studio [14]. In total, 1025 frames were used for training the deep learning models, 137 for validation, and 342 for testing their performance on frames the model has not encountered before, allowing comparison with annotated reference frames. Each video was fully assigned to either training, validation or testing set. Eight deep learning models underwent quantitative analysis to identify the most suitable one for real-time anatomy recognition in RAMIE. DeepLabv3 [15], DeepLabv3+ [16], PSPNet [17], and FPN [18] were included for their wide adoption in medical image analysis. Mask2Former [19], Segformer [20], Segmenter [21], and SegNeXt [22] were included for their use of attention mechanisms. These mechanisms potentially help focus on important image regions, improving segmentation performance under occlusions like blood or smoke during surgery. In contrast, models without attention treat all areas equally, reducing performance under occlusion. All trained models are made publicly available at: https://github.com/rlpddejong/SurgSegBench. While various metrics can be used to evaluate model performance, real-time anatomy segmentation should be both accurate and smooth from a surgical point of view. Therefore, all models were assessed using the Dice score, which quantifies spatial overlap on a scale from 0 to 1, the Average Symmetric Surface Distance (ASSD), which measures label margin accuracy in pixel distance. Both metrics were calculated using macro-averaging across classes, ensuring that each structure contributes equally to the final score. Additionally, we calculated frames per second (FPS) as an additional metric. A high Dice score and a low ASSD are desirable, and the higher the FPS the smoother the video.

To verify the dataset, inter-labeler agreement was assessed on 100 randomly sampled frames from the training, validation and test datasets, ensuring a representative sample. The expert blindly labeled these frames which were compared to the original frames, using the Dice similarity coefficient as a measure of global overlap to measure the quality of the labels.

### Dataset 2—surgical phases

Dataset 2 included 38 randomly selected RAMIE recordings from over 100 unlabeled videos. The annotation protocol was based on a previous study of Kingma et al. [23] that divided the thoracic part of the RAMIE procedure into 12 phases. To better match the anatomical area visible per phase, we combined the tracheoesophageal dissection and left recurrent laryngeal nerve dissection into one phase. This led to 11 surgical phases in our protocol: mobilization of the inferior pulmonary ligament (1), pericardial dissection (2), right pleural dissection (3), division of the azygos vein (4), superior mediastinal pleural dissection (5), right paratracheal dissection (6), tracheoesophageal and left recurrent laryngeal nerve dissection (7), para-aortic and mesoesophageal dissection (8), subcarinal lymph node dissection (9), aortopulmonary window lymph node dissection (10), and hiatal dissection (11). For each surgical phase, anatomical borders were predefined as posterior, anterior, cranial, and caudal borders. The borders were based on anatomical landmarks used by surgeons to safely perform the dissection within each phase. Two miscellaneous phases were added: non-standard (12) for transitions and out-of-the-ordinary actions, and camera out of body (13), in order to cover everything that can be seen during the video. The “boundaries” of a transition were the first and last frame of the camera moving from and to the borders of another phase. A detailed overview of the defined borders of the phases can be found in Table 1. Whenever the surgeon aims the camera within the predefined boundaries, the frames were allocated to the corresponding phase. As our aim of surgical phase recognition is to better understand the surgical scene to aid anatomy recognition, phase labels were allocated based on camera movement, which determines the visible anatomy in the scene, and not based on surgical actions which are known in related work [24]. Video labeling was done at 25 FPS using VirtualDub, assigning each frame to 1 of the 13 phases.

**Table 1:** Predefined borders of the surgical phases for the annotation protocol

Phase	Name	Posterior border	Anterior border	Cranial border	Caudal border
1	Mobilization inferior pulmonary ligament	N.A	N.A	End of pulmonary ligament	Start of pulmonary ligament
2	Pericardial dissection	Mediastinum	Right lung	Cranial border of right pulmonary vein/caudal border of right main bronchus	Diaphragm
3	Right pleural dissection	Mediastinum	Right lung	Caudal border of the arch of azygos vein	Cranial border of right pulmonary vein/caudal border of right main bronchus
4	Division arch of azygos vein	Azygos vein	Superior vena cava	Cranial border of the arch of the azygos vein	Caudal border of the arch of the azygos vein
5	Superior mediastinal pleural dissection	Vertebrae	Superior vena cava	Thoracic inlet	Cranial border of the arch of the azygos vein; or its previous location when the vein is clipped
6	Right paratracheal dissection	Right border of the trachea	Right lung/superior vena cava	Thoracic inlet	Caudal border of right main bronchus
7	Tracheoesophageal & left recurrent laryngeal nerve dissection	Esophagus	Right border of trachea	Thoracic inlet	Cranial border of carina
8	Para-aortic and mesoesophageal dissection	Azygos vein	Esophagus	Clipped azygos vein	Diaphragm
9	Subcarinal lymph node dissection	Right border of left main bronchus; or the esophagus	Left border of right main bronchus	Caudal border of carina	Cranial border of pulmonary veins
10	Aortopulmonary lymph node dissection	Caudal border of aortic arch	Left border of left main bronchus or the trachea	Caudal border of aortic arch	Crossing point of left main bronchus and aorta
11	Hiatal dissection	Vertebrae, azygos vein or aorta	Inferior vena cava	N.A	Diaphragm
12	Non-standard surgical actions and transitions	N.A	N.A	N.A	N.A
13	Camera out of body	N.A	N.A	N.A	N.A

Please note that the borders are based on the anatomical positions in the body and do not correlate with the location of what is seen in the frame or video (for example, the anatomical posterior border is the upper border in the camera view)

*N.A.* not applicable

Within this dataset, the videos were split based on patient level: 21 videos were used for training, 6 for validation and 11 for testing, using the same principle as for anatomy recognition. Several state-of-the-art models were selected for benchmarking based on their effectiveness with other surgical datasets. These models differ in how they process spatial and temporal information, which impacts their overall capability to recognize surgical phases. SV-RCNet [25] and TMRNet [26] are end-to-end architectures that integrate a ResNet-50 backbone with a Long Short-Term Memory (LSTM) module for joint spatial and temporal modeling. TMRNet extends SV-RCNet by introducing a memory bank fusion mechanism. In contrast, the remaining methods follow a two-stage paradigm, where a backbone network is first trained on a frame-by-frame basis, followed by a separate temporal modeling stage. TeCNO [27] is a multi-stage temporal convolutional network (MSTCN), whereas Causal-ASFormer proposed in [12] is a Transformer-based temporal modeling approach that employs causal attention to prevent access to future information. Trans-SVNet [28] further integrates temporal embeddings from TeCNO with spatial representations using Transformer blocks. All models are trained under a causal setting, ensuring that no future information is used during inference. Additionally, we investigate the use of pretrained models on surgical datasets [29], in particular DINO-based vision encoders. The DINOv2 ViT-Large model was selected due to its strong performance on related surgical benchmarks and was combined with both MSTCN and ASFormer temporal modules. The implemented models and code are publicly available at: https://github.com/Yipinggggg/SurgPhaseBench. For evaluation, frame-wise accuracy, balanced accuracy, overlap-based scores, and edit score were used following [30].

To assess inter-labeler agreement and quality control, two additional videos—excluded from the training, validation, and testing sets—were independently annotated by three blinded raters: an expert surgeon, a medical research fellow, and a technical research fellow (the two primary annotators of the dataset). Percentage of agreement was used, as well as Cohen’s Kappa (scale 0–1), which assesses the level of agreement between two observers, taking occurring by chance into account and providing a more accurate assessment of interobserver agreement.

## Results

### Dataset 1—anatomy and surgical tool recognition

Table 2 illustrates average model performance for anatomy recognition. It can be observed that the traditional CNNs achieve the highest frame rate of 87–100 FPS. In contrast, attention-based networks excel in terms of segmentation quality, with the highest Dice score reaching 0.72, versus 0.62 in the CNN group. SegNeXt, the best-performing model, performs at a frame rate of 25 FPS facilitating real-time deployment.

**Table 2:** Frames per second (FPS) and performance metrics for various models tested on the RAMIE anatomy segmentation dataset

Model	FPS	Dice score	ASSD [pixels]
DeepLabv3^a^	87	0.61 ± 0.16	22 ± 26
Deeplabv3+^a^	**100**	0.62 ± 0.16	20 ± 22
PSPNet^a^	96	0.62 ± 0.16	19 ± 18
FPN^a^	87	0.56 ± 0.15	23 ± 24
Mask2Former^b^	19	0.70 ± 0.16	16 ± 18
Segformer^b^	69	0.63 ± 0.16	20 ± 23
Segmenter^b^	30	0.69 ± 0.16	14 ± 17
SegNeXt^b^	25	**0.72 ± 0.15**	**13 ± 16**

^a^traditional CNN; ^b^attention-based network

Best performing scores are shown in bold

**Fig. 1:**
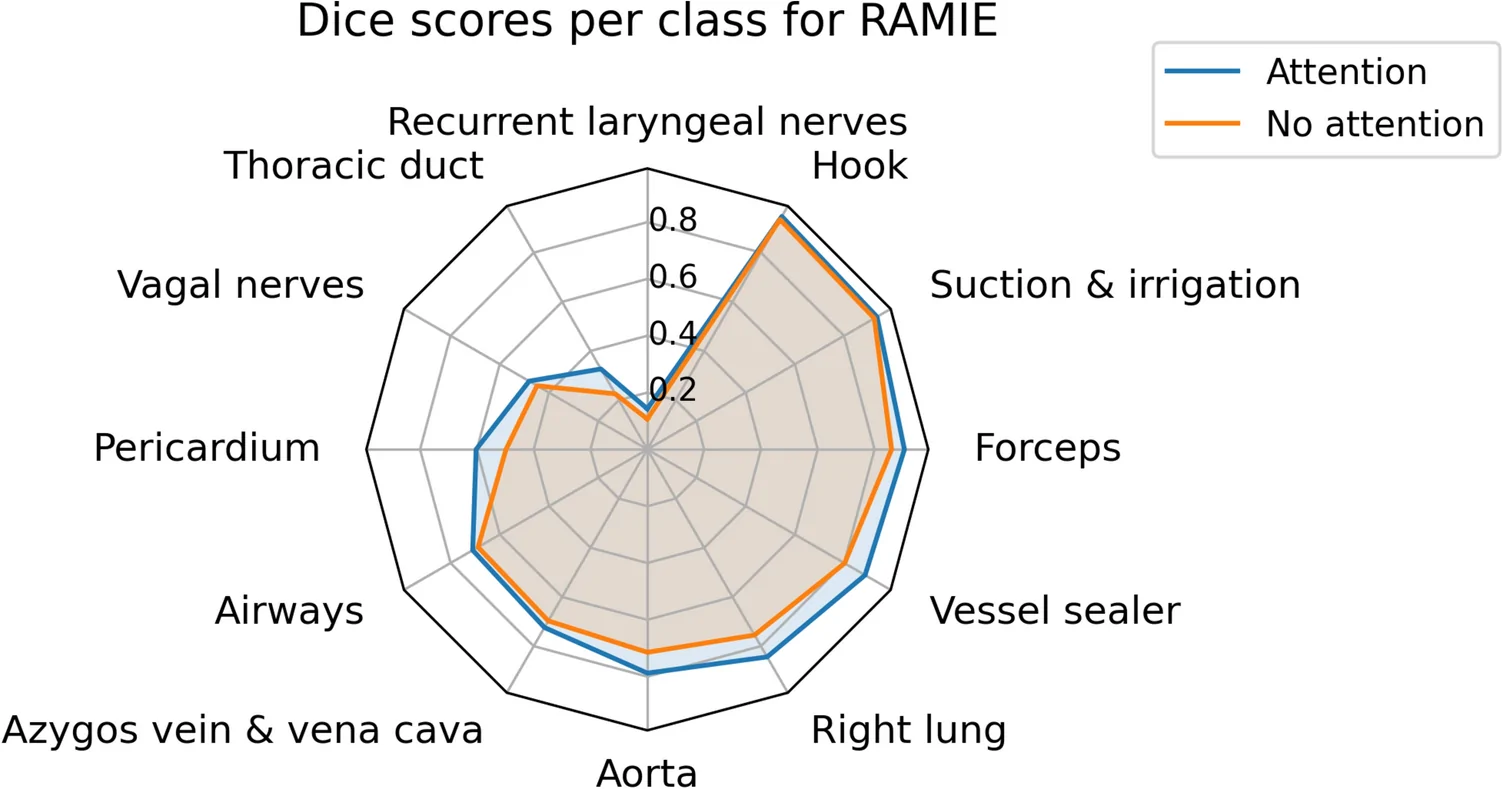
Performance of the different types of models on the RAMIE segmentation dataset, comparing attention and no-attention-based models in the Dice score per annotated class. The closer the Dice is to 1, the more accurate

Two types of trained models, with and without attention, were tested. Figure 1 compares model performance using Dice scores for each annotation class. Attention-based models consistently achieve higher scores across all twelve segmentation classes. Performance varies by class: surgical tools and larger anatomical structures, such as the lungs, show the highest scores, while smaller and underrepresented structures, like the thoracic duct and nerves, perform worse. Surgical tools and the aorta are well-segmented by all models.

**Fig. 2:**
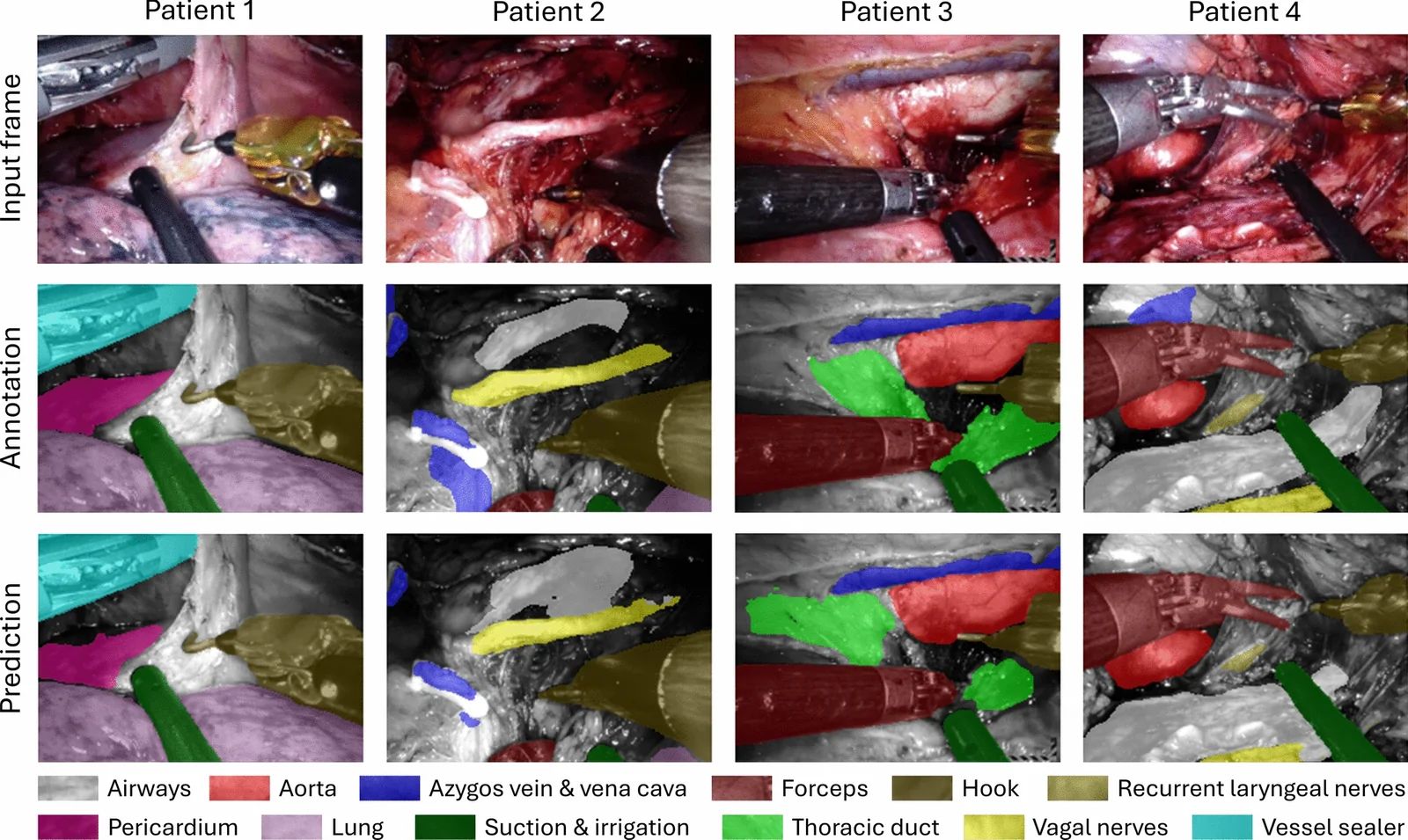
Visualization of anatomical structure predictions from the best-performing model across several patients. The top row contains input images, the middle row contains reference annotations, and the bottom row represents model predictions. Furthermore, a legend is included for all classes in the dataset

Figure 2 visualizes four RAMIE images where the best-performing model predicts anatomical classes. The visual examples demonstrate that the model detections generally align well with the reference annotations. However, the precise boundaries are not always accurately captured. For instance, in patient 2, the airways are overpredicted, while in patient 3, the thoracic duct is partially both under- and overpredicted. In patient 4, the azygos vein is not detected, likely due to partial occlusion by surgical instruments.

All classes were used to assess inter-labeler agreement in tissue and instrument labeling. Results are presented in Table 3. Overall, high agreement was observed, with most Dice scores above 0.80. The highest agreement was found for well-defined structures such as the aorta and lung, as well as surgical instruments (≈ 0.92–0.97). Moderate agreement was observed for the airways, azygos vein & vena cava, and pericardium (≈ 0.81–0.87). Lower agreement was seen for smaller and less distinct structures, including the recurrent laryngeal nerves, thoracic duct, and vagal nerves (≈ 0.58–0.79). These findings suggest that agreement is influenced by structure size, visibility, and boundary clarity.

**Table 3:** Inter-labeler agreement measured as Dice score between three independent labelers: the surgeon, technical PhD, and medical PhD

Class	Surgeon, technical PhD	Surgeon, medical PhD	Technical, medical PhD
Airways	0.81	0.84	**0.86**
Aorta	0.93	**0.94**	0.92
Azygos vein & vena cava	0.82	**0.86**	0.82
Forceps	**0.96**	0.95	0.95
Hook	0.96	**0.97**	0.96
Recurrent laryngeal nerves	0.68	**0.74**	0.71
Pericardium	**0.84**	0.81	0.83
Lung	0.92	**0.96**	0.93
Suction & irrigation	0.95	**0.97**	0.95
Thoracic duct	0.71	**0.79**	0.73
Vagal nerves	0.58	0.69	**0.70**
Vessel sealer	0.96	**0.97**	0.96
Mean	0.84	**0.87**	0.86

Best performing scores are shown in bold

### Dataset 2—surgical phase recognition

**Fig. 3:**
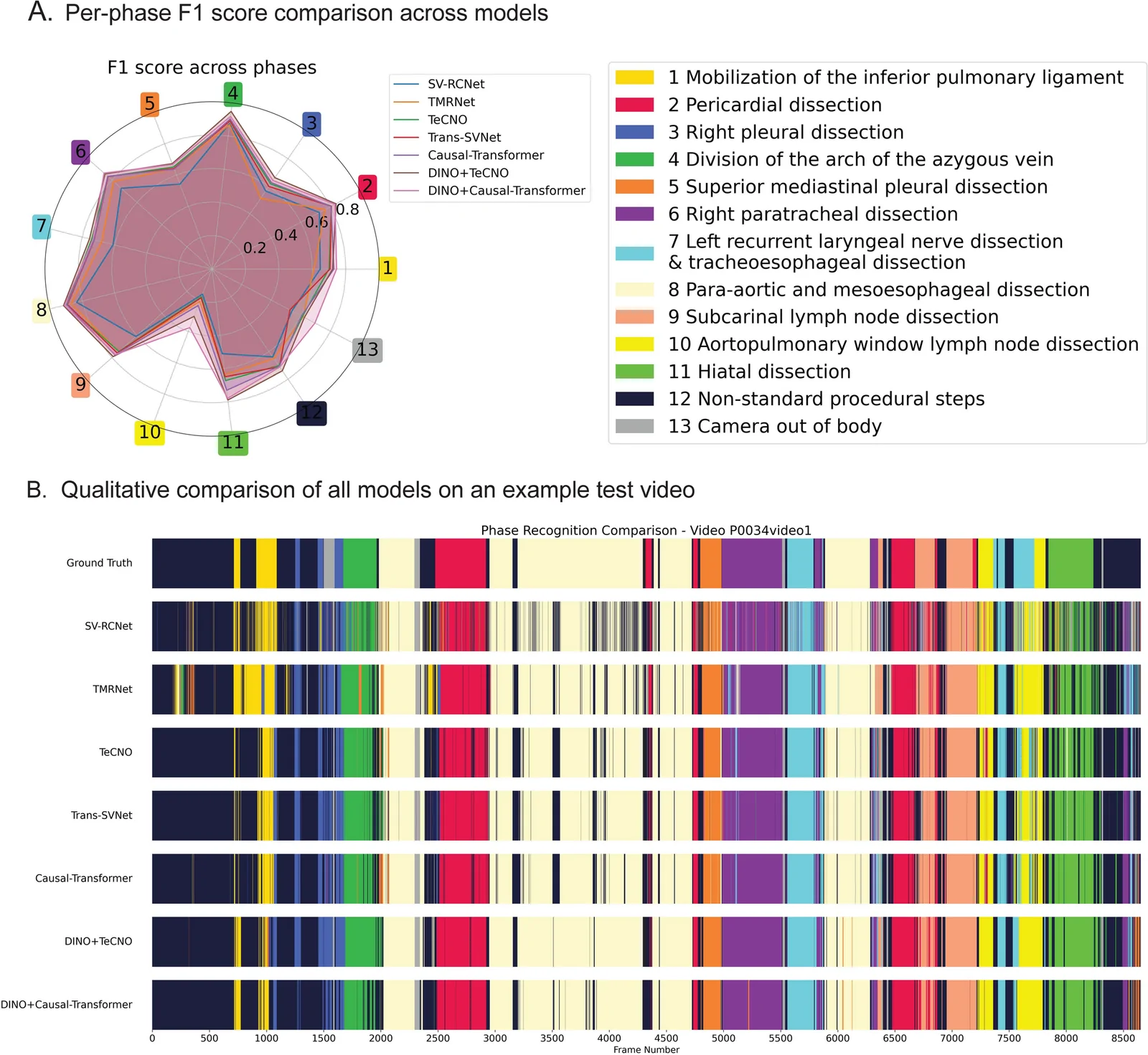
Surgical phases in RAMIE and the performance of different models on phase recognition within the RAMIE phase dataset. **A** per-phase F1 scores for each model, with the color legend for the thirteen surgical phases shown on the right; **B** qualitative results in one of the videos showing the ground truth in the top row, and seven comparative models in the rows below

Table 4 summarizes the performance of all benchmarked models, while Fig. 3A presents the per-phase results. Qualitative analysis in Fig. 3B revealed frequent misclassification between phase 10 (AP window) and phases 7 (tracheoesophageal and left laryngeal nerve dissection) and 9 (subcarinal dissection). Errors mainly occur near phase transitions, with phases 3 (right pleural dissection) and 10 (AP window) posing the greatest challenges.

**Table 4:** Performance for various models tested on the RAMIE surgical phase dataset

Model	Summary	Frame-wise accuracy	Balanced accuracy	F1@25	Edit score
SV-RCNet	ResNet50 + LSTM	71.63 ± 3.70	67.63 ± 3.25	3.87 ± 0.95	3.90 ± 0.69
TMRNet	ResNet50 + LSTM + memory bank fusion	75.75 ± 4.54	72.62 ± 4.83	15.11 ± 3.65	12.65 ± 3.04
TeCNO	ResNet50 + MSTCN	79.68 ± 4.51	73.18 ± 5.20	20.63 ± 2.62	14.95 ± 2.14
Trans-SVNet	ResNet50 + MSTCN + transformer variant	79.67 ± 4.12	73.68 ± 4.50	11.12 ± 1.09	8.69 ± 1.15
Causal-ASFormer	ResNet50 + transformer variant	80.06 ± 4.06	75.92 ± 4.99	20.88 ± 3.59	15.08 ± 2.99
DINO + TeCNO	Pretrained DINO ViT-Large + MSTCN	**82.81 ± 4.06**	**78.11 ± 3.47**	**31.10 ± 4.69**	**23.32 ± 3.15**
DINO + Causal-ASFormer	Pretrained DINO ViT-Large + transformer variant	81.66 ± 3.85	75.70 ± 2.90	30.40 ± 3.41	21.87 ± 2.31

Best performing scores are shown in bold

Mean ± standard deviation is computed across videos in the test set

**Fig. 4:**
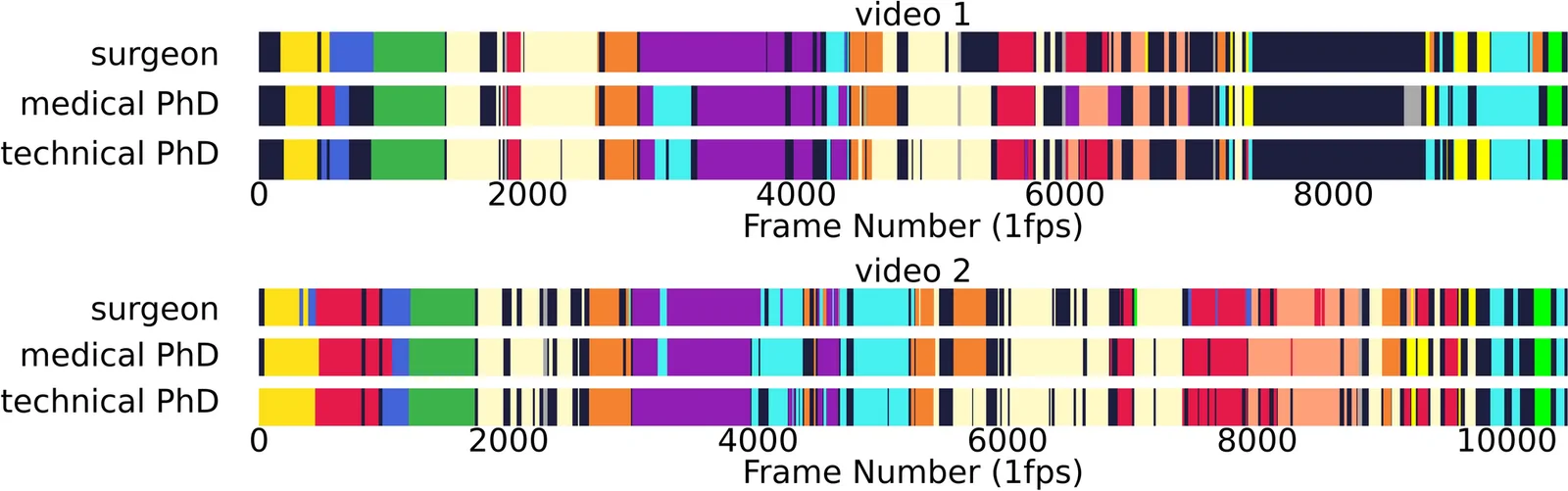
Results of inter-labeler agreement study. Two videos were blindly labeled by an expert surgeon, a medical PhD researcher, and a technical PhD researcher. Both were trained by the expert surgeon to identify surgical phases, although the technical researcher had less prior medical background and clinical exposure. Labeled phase sequence is shown per frame number, indicated by its assigned color as seen in Fig. 3A

To assess inter-labeler agreement, two dataset videos labeled by a technical research fellow were blindly relabeled by an expert surgeon and medical research fellow and compared. Cohen’s kappa and agreement percentages ranged from 0.70–0.82 to 74.89–83.83%, respectively. In Video 1, the technical fellow had the most accurate labels (79.56%), and in Video 2, the medical fellow (83.83%), while the technical fellow achieved 81.41%. Figure 4 visualizes the two videos, confirming correct phase order. Disagreement mainly occurred at phase starts/ends, during frequent transitions, and non-standard actions, which explained inter-labeler variation in anatomical areas defining the surgical phases.

## Discussion

In this study, we developed two of the most comprehensive RAMIE datasets to eventually facilitate intraoperative real-time anatomy and phase recognition. We evaluated multiple models to identify the most effective approaches, as well as to uncover current limitations.

All models perform well in segmenting surgical instruments due to their frequent occurrence and consistent appearance, as noted in recent literature [31]. This applies to the aorta, azygos vein, and right lung, which have distinct characteristics. Segmentation is poorer for airways and pericardium, as their boundaries are often obscured. Low accuracy labels may indicate inaccurate boundaries or false positives, potentially disturbing the surgeon. While no standard metric defines clinically acceptable accuracy, improvement is needed for underperforming classes. The lowest performing segmentations were the nerves and thoracic duct, which are hard to distinguish from surrounding tissue and appear infrequently. These structures are also challenging for surgeons in training, highlighting the need for more data from these classes [32]. Including prominent anatomical structures, such as the lung or azygos vein, is important even though they are easily recognized by the trained eye. Their inclusion will help improve algorithms for recognizing more challenging structures by providing a better understanding of the overall surgical landscape, particularly during zoomed-in field-of-view segments of RAMIE. Anatomical landmarks are crucial for surgeons as they provide orientation, boundaries and guidance, especially in oncologic surgery regarding radicality and lymphadenectomy. As the mediastinum is a narrow space, almost all anatomical landmarks that are used by surgeons during RAMIE [33] were included in our dataset. The mesoesophagus was not included as distinct recognition by the algorithm from other connective tissue was not expected to be feasible due to similar coloring because of the fatty tissue and its less distinct delineated boundaries. The diaphragm, vertebrae and thoracic aperture were not included because of the low added value as they are easily recognizable due to their fixed location and provide limited surgical guidance. The careful selection of included structures led to a complex and sophisticated dataset. Further advancements are needed to address the clinical need for high-quality recognition of structures like nerves and the thoracic duct. Ultimately, these improvements will contribute to the development of augmented reality applications, such as overlaying a preoperative, patient-specific 3D model to enhance intraoperative navigation and orientation. The results for the azygos vein, aorta and lung were comparable to a previous study [10], although their analysis was based on frames with less obstruction, whereas our dataset includes a wider range of frames from full thoracic dissection. Two studies investigated recurrent laryngeal nerve recognition in RAMIE; one achieved a Dice score of 0.58 [34], while the other showed lower accuracy, using a different metric that makes comparison difficult, but demonstrated that AI significantly improved surgeons’ recognition of the right recurrent laryngeal nerve [35].

The most accurate model, SegNeXt, excels at overlap and precise boundary delineation, with minimal distance between the predicted and reference boundaries, as reflected in its low ASSD. This makes it well-suited for anatomy recognition in RAMIE. With a frame rate of 25 FPS, it is generally acceptable for surgical applications, ensuring no significant delays in real-time detection that would affect the surgeon.

As for surgical phase recognition, the RAMIE phase dataset is complex due to the narrow mediastinal space and variability in phase sequences. Phase sequence variability depends on the surgeon’s preference, habits, patient anatomy, and oncologic disease [36]. The best model achieved 82.8% accuracy in phase recognition, indicating that both strong backbone and temporal modeling are required for optimal performance. Similar to our anatomy and surgical tool dataset, our surgical phase dataset shows a comparable performance to other studies while increasing the complexity of the task. Takeuchi et al. [37] reported 84% accuracy, using a protocol with nine phases and larger anatomical regions. Upon closer evaluation, we found that classification errors occur mostly at phase transitions, where delineating cut-off frames remains challenging, particularly when key anatomical structures are not yet visible. Additionally, current metrics are inadequate for assessing phase transition detection accuracy, highlighting a need for further exploration. Surgical phase recognition could support surgical scene understanding and recognition of related anatomical structures and surgical tools used. Future work will aim to use surgical phase recognition to improve anatomy recognition accuracy. Our surgical phase dataset is based on the standardization of RAMIE, leading to a complex and clinically relevant dataset. It could facilitate future analysis of phase duration or sequence, which can be used for training, educating, and improving efficiency [24].

An analysis of label frequency in both datasets revealed an imbalance in the representation of certain class categories (tissues or phases). In dataset 1, which focuses on anatomical structures, certain structures appear only during specific phases of the surgery. The varying sizes of these structures also affect the proportion of annotated pixels per structure. In dataset 2, some surgical phases are significantly shorter than others, resulting in uneven representation of phases. In both datasets, this imbalance leads to certain classes being underrepresented when sampled randomly. To address this issue, a more diverse and extensive set of training data is required.

Acquiring datasets for deep learning is challenging due to the limited availability of experts and time-consuming annotations. As a result, both datasets were primarily labeled by non-expert research fellows. Our inter-labeler agreement study of anatomy segmentation revealed mislabeling of challenging tissue boundaries, highlighting the need for additional training to align better with expert annotations. While the research fellows produced accurate segmentations for identified structures, improving recognition and minimizing missed segmentations is key to achieving expert-level performance. The inter-labeler agreement study of surgical phase recognition showed high accuracy (80%+), with accuracy loss mainly due to unclear cut-off points, which could be improved with refined labeling protocols. Future work will focus on improving, diversifying, and expanding both datasets, as well as combining algorithms for surgical phase recognition and anatomy segmentation to compare accuracy.

## Conclusion

This study presents two new datasets for labeled anatomy and tools, and surgical phase recognition in RAMIE, alongside an evaluation of deep learning algorithms for anatomical structure and surgical tool detection and surgical phase classification. To our knowledge, the datasets are the most comprehensive and complex for RAMIE while our results show similar or improved performance compared to literature. These contributions provide a foundation for the future development of computer-aided surgical assistance. Our evaluation demonstrates that accurate anatomical recognition can be achieved at speeds acceptable for real-time surgical use, with overall surgical phase recognition reaching 82.8% accuracy. Frequently in-view and stable structures were reliably identified in real-time, while more complex ones remain challenging. These results confirm the potential of deep learning models to enhance surgical navigation and safety in RAMIE. Furthermore, this study lays the foundation for future advancements, representing a crucial step toward transforming this complex surgical procedure with real-time, AI-driven support.

## Supplementary Information

Below is the link to the electronic supplementary material.

Supplementary file1 (DOCX 26 kb)

## Data Availability

The datasets generated and analyzed during the current study are not publicly available.
