# Supplementary material for: Automatic recognition of anatomical structures and surgical phases in robot-assisted minimally invasive esophagectomy (RAMIE) using deep learning: a retrospective cohort study
Source: Surg Endosc. 2026 Jul 9;40(9):7772–82. doi: 10.1007/s00464-026-12985-1 (PMC13582270; doi:10.1007/s00464-026-12985-1)
Supplement: Supplementary file 1 [file 464_2026_12985_MOESM1_ESM.docx]

**Appendix**

**General specifications**

All experiments were conducted on a NVIDIA GeForce RTX 2080 Ti GPU (NVIDIA Corp., CA, USA). All videos were recorded at a frame rate of 25 Hz with a resolution of 960x540 pixels, and their average duration after editing was two hours. Black borders and the graphical user interface were removed resulting in a final resolution of 668x502.

**Anatomy recognition**

Eight state-of-the-art deep learning models were selected for comparison and were pretrained to reach higher accuracy without including additional annotated data. Pretraining was done on ADE20k [38], which is aimed at semantic segmentation. Frames from our dataset were augmented to expose to model to different looking inputs, which improve model performance and robustness. Examples of augmentations used were horizontal or vertical flip, blurring, brightness, contrast and rotation. All models were assessed using the Dice score, the Average Symmetric Surface Distance (ASSD) and frames per second (FPS). All metrics were calculated on a per-frame basis, and statistical significance was assessed using a Wilcoxon signed-rank test.

**Surgical phase recognition**

Metrics used for measuring the accuracy of surgical phase recognition:

- Accuracy = number of correctly identified frames/total number of frames
- Precision = number of correctly identified frames in a phase/total number of frames identified by AI in phase
- Recall = number of correctly identifies frames in a phase/total number of frames in a phase
- Jaccard = number of frames correctly identified in both prediction and ground truth/total number of frames
- F1 = (2 recall * precision)/(recall+precision)

Precision (the positive predictive value), recall (sensitivity) and Jaccard (quantifying similarity between prediction and ground truth) were first calculated for each phase, then averaged over all phases. Using precision and recall, the F1 score was calculated, which provides insights into the model’s performance by the count of true positives on a scale of 0 to 1, a score closer to 1 indicates a robust model.
